# Perioperative immunotherapy for esophageal squamous cell carcinoma

**DOI:** 10.3389/fimmu.2024.1330785

**Published:** 2024-02-19

**Authors:** Dan D. Wei, Jin M. Fang, Huan Z. Wang, Jian Chen, Shuai Kong, Yan-Yi Jiang, Yuan Jiang

**Affiliations:** ^1^ Esophageal and Gastrointestinal Tumor Center, Hefei Cancer Hospital, Chinese Academy of Sciences, Hefei, China; ^2^ Anhui Province Key Laboratory of Medical Physics and Technology; Institute of Health and Medical Technology, Hefei Institutes of Physical Science, Chinese Academy of Sciences, Hefei, China; ^3^ Science Island Branch of Graduate School, University of Science and Technology of China, Hefei, China

**Keywords:** esophageal squamous cell carcinoma, immunotherapy, chemotherapy, immune checkpoint blockade, PD1/PD-L1

## Abstract

Esophageal squamous cell carcinoma (ESCC) is the main prevalent histological subtype and accounts for 85% of esophageal cancer cases worldwide. Traditional treatment for ESCC involves chemotherapy, radiotherapy, and surgery. However, the overall prognosis remains unfavorable. Recently, immune checkpoint blockade (ICB) therapy using anti-programmed cell death-1 (PD-1)/PD-1 ligand (PD-L1) antibodies have not only achieved remarkable benefits in the clinical management of ESCC but have also completely changed the treatment approach for this cancer. In just a few years, ICB therapy has rapidly advanced and been added to standard first-line treatment regimen in patients with ESCC. However, preoperative immunotherapy is yet to be approved. In this review, we summarize the ICB antibodies commonly used in clinical immunotherapy of ESCC, and discuss the advances of immunotherapy combined with chemotherapy and radiotherapy in the perioperative treatment of ESCC, aiming to provide reference for clinical management of ESCC patients across the whole course of treatment.

## Highlights

- The combination of immunotherapy and chemotherapy offers a potential perioperative option for patients with resectable ESCC.
- Neoadjuvant immunotherapy combined with chemotherapy results in encouraging major pathologic response rates without introducing new adverse reactions in patients with operable ESCC.

## 1 Introduction

Esophageal cancer ranks among the most common malignant tumors, and causes more than half a million cancer-related mortality world-wide each year (1). Esophageal squamous cell carcinoma (ESCC) and esophageal adenocarcinoma are the two main pathological subtypes of esophageal cancer, and have almost completely distinct biologic, geographic and etiologic characteristics. ESCC accounts for about 85% of the 604,100 incident esophageal cancers each year, predominating in regions of Eastern Asia and Africa (2). Traditional treatment options for ESCC include chemotherapy, radiotherapy, and surgery. However, due to the neglect of early symptoms of ESCC, many patients are diagnosed at an advanced stage, leading to a poor overall prognosis with a five-year survival rate of approximately 10%20% (3). Surgery combined with neoadjuvant chemoradiotherapy is the preferred treatment for locally advanced ESCC patients, but recurrence and metastasis still occur in many patients after treatment (4).

Recent years, cancer immunotherapy has emerged as a promising anti-tumor therapeutic strategy after surgery, radiotherapy and chemotherapy. In particular, immune checkpoint blockade (ICB) antibodies have been developed to enhance immune responses or/and alleviate immunosuppression by acting on immune checkpoints such as cytotoxic T lymphocyte antigen 4 (CTLA-4), programmed cell death-1 (PD-1) or PD-1 ligand (PD-L1) PD-1, and PD-L1. Currently, the most advanced ICB agents are anti-PD-1/PD-L1 antibodies because of the central role of T cells in tumor immunological surveillance, which have demonstrated substantial clinical progress (5). However, the efficacy of immunotherapy alone is only 20%-40% (6). Limited overall responses and the lack of reliable biomarkers predicting patients response are major obstacles to immunotherapy. This reflects the compelling need of unveiling novel targets for immunotherapy that allow to expand the spectrum of ICB-based strategies to achieve optimal therapeutic efficacy and benefit for cancer patients (7). New immune checkpoint inhibitors that mediate T cell inhibitory signaling such as lymphocyte-activation gene 3 (LAG-3), T-cell immunoglobulin and mucin domain-3 (TIM-3), V-domain Ig suppressor of T cell activation (VISTA), T-cell immunoreceptor with Ig and ITIM domains (TIGIT), inducible T-cell co-stimulatory receptor (ICOS), Nuclear receptor subfamily 2, group F, member 6 (NR2F6), sialic acid-binding immunoglobulin-like lectins-8 (SIGLEC8) and B and T lymphocyte attenuator (BTLA) are developed to overcome resistance to cancer immunotherapy and improve the outcome for cancer patients (8–10). Some of these new drugs and therapeutic regimens have been tested in clinical studies and achieved promising results.

In clinical practice, immune combined chemotherapy is mostly recommended to enhance the tumor response rate. Herein, we summarize the progress of immunotherapy combined with chemotherapy in the perioperative treatment of ESCC patients, along with proposing possible challenges and future solutions for the current implementation of perioperative immunotherapy in ESCC.

## 2 ICBs targeting PD-1/PD-L1

**Figure 1:**
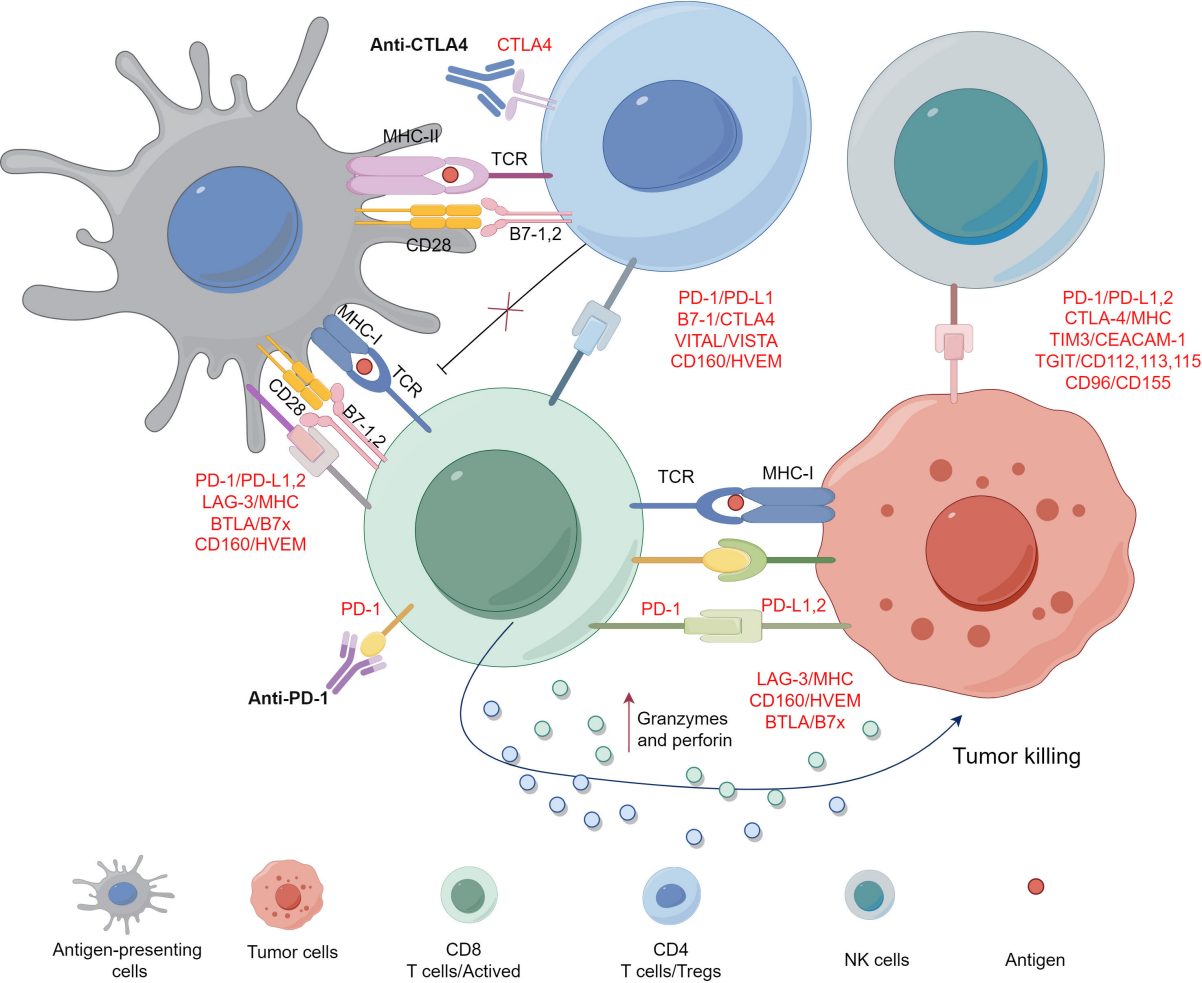
PD-1/PD-L1 interaction between T cell and tumor cell.

PD-1/PD-L1 represents a common immune checkpoint in T cell activation, with PD-1 being expressed on the surface of T cells and PD-L1 often expressed in tumor cells except for macrophages (11). The binding of PD-1 to its ligand PD-L1 activates an intercellular inhibitory signaling pathway of T cells, resulting in the inhibition of T cell function. Furthermore, activated T cells can express CD80 as a receptor for transmitting inhibitory signals, leading to the tolerance of peripheral T cell. PD-L1 expression by tumor cells can evade attack by CD8+ T cells (12). When cancer cells release tumour-associated neoantigens, these neoantigens will be recognized by antigen-presenting cells and rendered to immune cells by the interaction with T cell receptor (TCR) (13). CD8^+^ T cells will be activated and release Granzyme and Perforin to kill cancer cells. Conventionally, PD-L1 or PD-L2 express on the surface of antigen presenting cells to prevent autoimmune disorders. However, tumor cells acquire this skill and escape immune attack successfully, knowing as immune evasion by upregulation of immunoinhibitory molecules (e.g., PD-1, CTLA-4, TIM3, TIGIT, CD96), or their ligands (14). Blocking the interaction between tumor and T cells such as PD-1/PD-L1 or CTLA-4/B7 axis with immune checkpoint inhibitors could avoid immune evasion and eliminate malignant cells. (**Figure 1**). Anti-PD-1/PD-L1 monoclonal antibody reactivates the immune response of T cells to tumors by blocking the binding of PD-1 to PD-L1 protein, thereby achieving an antitumor effect (15). Immune checkpoints play a complex balance role in the body, maintaining self-tolerance and regulating the immune response. The stimulation pathway promotes T cell activation, while the inhibition pathway inhibits T cell activation. Anti-PD-1/PD-L1 antibodies are currently the most extensively investigated immune checkpoint inhibitors and have been recommended for treating various of malignancies (**Table 1**).

**Table 1:** Immunotherapies in the clinic.

Agent	Target	Tumor type	Clinical indication being evaluated	Groups in the trial and the treatment (dosing regiment and dosage level)	Result	Reference
Nivolumab	PD1	ESCC	CheckMate 648	Nivolumab plus chemotherapy or ipilimumab vs chemotherapy	mOS: 15.4 months vs 9.1 months	(16)
		Non-small-cell lung cancer	CheckMate 078	nivolumab vs docetaxel	mOS: 11.9 months vs 9.5 months	(17)
		Head and neck squamous cell carcinoma	CheckMate 141	Nivolumab vs chemotherapy	mOS: 7.7 months vs 3.3 months	(18)
		Stomach cancer	ATTRACTION-2	Nivolumab vs placebo	mOS: 5 .3 months vs 4.1 months	(19)
		Uroepithelial carcinoma	CheckMate 274	Nivolumab vs placebo	DFS: 22.0 months vs 10.9 months	(20)
		Malignant pleural mesothelioma	CheckMate 743	Nivolumab plus ipilimumab vs chemotherapy	mOS: 18.1 months vs 14.1 months	(21)
Pembrolizumab	PD1	ESCC (12)	KEYNOTE-181	Pembrolizumab vs chemotherapy	mOS: 7.1 months vs 6.9 months	(22)
		Melanoma	KEYNOTE-006	Pembrolizumab vs ipilimumab	mOS: 32.7 months vs 15.9 months	(23)
		Non-small cell lung cancer	KEYNOTE-091	Pembrolizumab vs placebo	mOS: 53.6 months vs 42.0 months	(24)
		Head and neck squamous cell carcinoma	KEYNOTE-048	Pembrolizumab alone or with chemotherapy versus cetuximab with chemotherapy	mOS: 13.0 months vs 10.7 months	(25)
		Colorectal cancer	KEYNOTE-177	Pembrolizumab vs chemotherapy	mPFS: 16.5 months vs 8.2 months	(26)
		Hepatocellular carcinoma	KEYNOTE-240	Pembrolizumab vs placebo	mOS: 13.9 months vs 10.6 months	(27)
		Triple-negative breast cancer	KEYNOTE-355	Pembrolizumab with chemotherapy vs chemotherapy	mOS: 23 months vs 16.1 months	(28)
Toripalimab	PD1	ESCC	JUPITER-06	Toripalimab plus chemotherapy vs placebo plus chemotherapy	mPFS: 5.7 months vs 5.5 months; mOS: 17.0 months vs 11.0 months	(29)
		Nasopharyngeal Carcinoma	JUPITER-02	Toripalimab plus chemotherapy vs placebo plus chemotherapy	mPFS: 11.7 months vs 8.0 months	(30)
		Uroepithelial carcinoma	POLARIS-03	Toripalimab	ORR:27.6% DCR:51.3%	(31)
		Non-small cell lung cancer	CHOICE-01	Toripalimab plus chemotherapy vs placebo plus chemotherapy	mPFS: 8.4 months vs 5.6 months OS: NR vs 17.1 months	(32)
Sintilimab	PD1	ESCC	ORIENT-15	Sintilimab plus chemotherapy vs chemotherapy	OS: 16.7 months vs 12.5 months PFS: 7.2 months vs 5.7 months	(33)
		Non-small cell lung cancer	ORIENT-11	Sintilimab plus chemotherapy vs chemotherapy	mPFS: 9.2 months: 5.0 months; mOS: 24.2 months vs 16.8 months	(34)
		Hodgkin lymphoma	ORIENT-1	Sintilimab	ORR:80.4% CR:34%	(35)
Camrelizumab	PD1	ESCC	ESCORT-1	Camrelizumab plus chemotherapy vs placebo + chemotherapy	mOS: 15.3 months vs 12.0 months; mPFS: 6.9 months vs 5.6 months; ORR: 72.1% vs 62.1%; mDoR, 7.0 months vs 4.6 months	(36)
		Non-small cell lung cancer	Camel02	Camrelizumab plus chemotherapy vs chemotherapy	mOS11.3 months vs 8.3 months	(37)
		Hepatocellular carcinoma	CARES-310	Camrelizumab plus rivoceranib vs sorafenib	mOS5.6 months vs 3.7 months	(38)
		Gastroesophageal Junction Adenocarcinoma	Ahead-G208	Camrelizumab combined with rivoceranib and chemotherapy versus chemotherapy	pCR:18.3% vs. 5.0%	(39)
Tislelizumab	PD1	ESCC	RATIONALE-302	Tislelizumab vs chemotherapy	mOS8.6 months vs 6.3 months	(40)
		nasopharyngeal cancer	RATIONALE-309	Tislelizumab chemotherapy vs placebo + chemotherapy	PFS:9.6 months VS 7.4 months; mOSNR vs 23 months	(41)
		Uroepithelial carcinoma	RATIONALE 311	Tislelizumab chemotherapy vs placebo + chemotherapy	mOS: 21.4 months vs 14.3 months	(42)
		small cell lung cancer	RATIONALE 312	Tislelizumab chemotherapy vs placebo + chemotherapy	mO: 15.5 months vs 13.5 months	(43)
		Non-small cell lung cancer	RATIONALE 304	Tislelizumab chemotherapy vs placebo + chemotherapy	mOS: 21.6 months vs 14.9 months	(44)
		Gastroesophageal Junction Adenocarcinoma	RATIONALE 305	Tislelizumab chemotherapy vs placebo + chemotherapy	mOS: 17.2 months vs 12.6 months	(45)
Durvalumab	PD-L1	Non-small cell lung cancer	PACIFIC	Adebrelimab+ chemotherapy vs placebo + chemotherapy	mOS: 15.3 months vs 12.8 months	(46)
		Small cell lung cancer	CASPIAN	Durvalumab vs placebo	PFS: 17.2 months vs 5.6 months; mOS: 47.5 months vs 29.1 months	(47)
Atezolizumab	PD-L1	Non-small cell lung cancer	IMpower132	atezolizumab + carboplatin or cisplatin + pemetrexed	PFS: 7.6 months vs 5.2 months mOS: 18.1 months vs 13.6 months	(48)
		Small cell lung cancer	IMpower133	carboplatin and etoposide with either atezolizumab or placebo	PFS: 5.2 months vs 4.3 months mOS: 12.3 months vs 10.3 months	(49)
		Hepatocellular carcinoma	IMbrave150	Atezolizumab plus Bevacizumab vs Sorafenib	mOS: 19.2 months vs 13.4 months; ORR: 30% vs 11%	(50)
Sugemalimab	PD-L1	Non-small cell lung cancer	GEMSTONE-302	Sugemalimab plus chemotherapy vs placebo plus chemotherapy	PFS: 7.8 months vs 4.9months; mOS: NR vs 14.75 months	(51)

ORR, overall response rate; DCR, disease control rate; PFS, progression-free survival; OS, overall survival; NA, not available/applicable; NC, nonacral cutaneous; PCR, Pathologic Complete Response.

In recent years, the in-depth study of immunotherapy has ushered ESCC into the era of immunotherapy. Pembrolizumab combined with chemotherapy has gained approval as a first-line treatment for unresectable locally advanced or metastatic esophageal or gastroesophageal junction cancer based on KEYNOTE-590 study (52). This combination is the first approved immunotherapeutic agent for ESCC. Subsequently, several phase III, randomized, multicenter studies in advanced ESCC have demonstrated promising clinical activities. For instance, studies of CheckMate 648 for nivolumab (16), KEYNOTE-181 for pembrolizumab (22), JUPITER-06 for toripalimab (29), ORIENT-15 of sintilimab (33), ESCORT-1st for camrelizumab (36), and RATIONALE-302 for tislelizumab (40) have confirmed the efficacy advantage of immunotherapy in treating advanced ESCC. These drugs have thus been recommended in clinical guideline as first- and second-line treatment options in advanced-stage ESCC. However, the benefit of perioperative immunotherapy especially in the neoadjuvant setting is still being exploring.

## 3 Research progress of neoadjuvant immunotherapy combined with chemotherapy for patients with resectable ESCC

As cancer research progresses, cancer diagnosis and treatment have embraced the multi-disciplinary treatment model (MDT). The therapeutic benefits of immunotherapy at various stages of tumor progression have gradually been recognized, although neoadjuvant immunotherapy in ESCC is still rarely investigated. Traditional neoadjuvant chemotherapy aims to minimize tumor lesions and achieve preoperative downstaging for radical surgery, while neoadjuvant immunotherapy can eliminate micro metastatic tumor lesions by enhancing anti-tumor immune response (53). Furthermore, receiving immunotherapy at the initial diagnostic stage occurs when the patient has relatively competent immunity, allowing for activating immune response. Thus, it is theoretically feasible to incorporate immunotherapy in the preoperative neoadjuvant or conversion treatment stage.

The initial exploration of neoadjuvant modality in the perioperative setting of esophageal cancer was CROSS study (54). This study confirmed that neoadjuvant chemoradiotherapy before surgery provided an overall survival (OS) benefit and which persisted for at least 10 years for patients with locally advanced resectable esophageal or gastroesophageal junction cancer. The CROSS study has thus become a landmark trial in the perioperative treatment of esophageal cancer. However, clinical practice has found that patients who underwent surgery after neoadjuvant chemoradiotherapy had a higher risk of postoperative complications such as esophageal fistula and bleeding, which also increased the complexity of the surgery. Previous studies have demonstrated that platinum-based chemotherapeutic agents promoted the expression of PD-L1 on tumor cells, and consequently exhibited a synergistic effect with anti-PD-L1 immunotherapy (55). The application of neoadjuvant immunotherapy in esophageal cancer first emerged from a single-center, prospective, single-arm PALACE-1 study (56), which aimed to assess the effectiveness and safety of preoperative pembrolizumab combined with chemoradiotherapy in the neoadjuvant treatment of locally advanced ESCC. The findings demonstrated that pembrolizumab combined with neoadjuvant concurrent chemoradiotherapy achieved a pathological complete response (pCR) rate of 55.6% (10/18), a major pathological response (MPR) rate of 89% (16/18), and a margin-negative R0 (R0 defined as no vital tumor present within 1 mm of the proximal, distal, or circumferential resection margins) resection rate of 94%. The KEYSTONE-001 study (57), conducted by Tianjin Medical University Cancer Institute & Hospital is the first research in the world to investigate the efficacy and safety of pembrolizumab combined with paclitaxel and cisplatin for neoadjuvant treatment of locally advanced ESCC. The latest findings indicated that among 29 patients who underwent surgery, a pCR rate of 41.4% (12/29), a MPR rate of 72.4% (21/29), and a R0 resection rate of 100% were achieved. These data support a favorable safety and prominent anti-tumor activity for anti-PD-1 immunotherapy combined with chemotherapy in patients with locally advanced resectable ESCC in the neoadjuvant setting. Another study called ESPRIT investigated the effectiveness and safety of neoadjuvant camrelizumab combined with chemotherapy in ESCC (58). Preliminary findings demonstrated that among 11 patients who received surgical treatment, the pCR rate was 45.5% (5/11), which was not associated with the expression of PD-L1, and lymph nodes alone (pT0) rate was 54.5% (6/11), with an R0 resection rate of 100%. The combination of camrelizumab, paclitaxel, and carboplatin as a neoadjuvant therapy regimen demonstrated good tolerability and achieve preoperative downstaging. The KEEP-G 03 study (59) assessed the feasibility and safety of neoadjuvant treatment of resectable ESCC with sintilimab plus triplet chemotherapy (liposomal paclitaxel, cisplatin, and S-1) every 3 weeks for two cycles. Of these 30 patients received surgical treatment, the MPR, pCR rates were 50.0% (15/30; 95% CI 33.2 to 66.9) and 20.0% (6/30; 95% CI 9.5 to 37.3), respectively. R0 resection rates were 100%. In the phase II TD-NICE study (60), 45 resectable ESCC patients were enrolled for 3 cycles of neoadjuvant tislelizumab combined with chemotherapy. Among them, 36 patients underwent surgery, the R0 resection, MPR and pCR rates were respectively 80.5%, 72% (25/36), and 50% (18/36). This study further proposed for the first time that tumor proportion score cut-off>1 was a better indicator to predict pCR than combined proportion score, and it was also confirmed that HLA-A02 gene mutation may be associated with pCR through next-generation sequencing. Additionally, TP53 (86%), NOTCHI (40%), FAT1(26%), CDKN2A (23%), and EP300(17%) were identified as the most mutated genes; however, no clear correlation between these genes and pCR or MPR was revealed. In summary, these studies suggest that immunotherapy combination with chemotherapy is a promising regimen with favorable efficacy and safety for the neoadjuvant treatment of ESCC.

## 4 Research progress of immunotherapy combined with chemotherapy in the adjuvant setting of ESCC

Adjuvant therapy aims to eliminate subclinical metastatic lesions and reduce the recurrence and metastasis rates (61). However, controversies still exist regarding adjuvant treatment for esophageal cancer in clinical practice. The 5-year survival rate with surgery alone for locally advanced esophageal cancer (pT3-4 or pN+) is only 20%40% (62). In the CheckMate577 study (63), the patients were randomly assigned into a 2:1 ratio to receive either nivolumab or placebo from 4 to 16 weeks after neoadjuvant radiotherapy and surgery. It was demonstrated that in patients who did not achieve clinical pCR to neoadjuvant immunotherapy in combination with chemotherapy following surgery, adjuvant nivolumab substantially prolonged median disease-free survival in the intention-to-treat population (20.8 months vs 10.8 months). Moreover, adjuvant nivolumab provided survival benefits regardless of histological type and pathological feature of lymph node, and no major differences were observed on safety in the patients receiving either adjuvant immunotherapy or not. A phase II randomized controlled study from Korea (64) assessed the effectiveness of adjuvant durvalumab after neoadjuvant concurrent chemoradiotherapy and surgery in 86 ESCC patients, with a median 38.7 months follow-up for survival analysis. Median disease-free survival (DFS) was not reached in any group. At 12, 24, and 36 months, the disease-free survival rates were 71%, 58%, and 55% in the durvalumab group and 73%, 61%, and 61% in the placebo group, respectively. No significant differences in disease-free survival and OS were observed between the durvalumab and placebo groups. However, this study initially indicated that PD-L1 expression after concurrent chemoradiotherapy could predict survival benefit in patients receiving adjuvant durvalumab after neoadjuvant concurrent chemoradiotherapy, although this finding needed further validation from larger randomized controlled studies. AIRES is a multicenter phase III trial in ESCC patients in China, aimed to assess the effectiveness and safety of tislelizumab combined with chemotherapy versus adjuvant tislelizumab alone in patients with radical resection for high-risk ESCC. The study is ongoing, and further better clinical data are anticipated.

Currently, the option of adjuvant therapy for esophageal cancer depends more on severity of the disease and local clinical practice models. The traditional belief is that adjuvant therapy may only be beneficial for patients with positive lymph nodes or an increased risk of recurrence, and the National Comprehensive Cancer Network (NCCN) previously recommended its use for patients who underwent incomplete resection (65). In 2021, Food and Drug Administration has approved nivolumab as an adjuvant treatment option for esophageal cancer patients received triple therapy based on the findings of the Checkmate577 study. The nutritional status of patients often deteriorates due to the reconstruction of the digestive system in esophageal cancer patients after surgery, and this issue is particularly prominent in Chinese patients with esophageal cancer. Many patients are often unable to tolerate traditional platinum-containing doublet chemotherapy after surgery, but they have a good tolerance to immune drugs, which makes adjuvant immunotherapy worthy of more in-depth research. From the initial CROSS trial to the Checkmate577 study, numerous curative possibilities have emerged for locally advanced resectable esophageal cancer. These studies also provide valuable references for the clinical managements of esophageal cancer.

## 5 Discussion

In recent years, there has been controversy for the treatment modalities of esophageal cancer in the perioperative setting (66), mainly focusing on neoadjuvant therapy. Current neoadjuvant therapy modalities include chemotherapy, chemoradiotherapy, immunotherapy, and immunotherapy combined with radiotherapy or chemoradiotherapy. In North America, neoadjuvant chemoradiotherapy followed by esophagectomy is the standard treatment option for locally advanced esophageal cancer. In Asia, neoadjuvant chemotherapy or neoadjuvant immunotherapy combined chemotherapy is more commonly conducted. We believe that the findings in clinical studies will provide clear references for the neoadjuvant treatment mode of esophageal cancer. Currently, neoadjuvant treatment for locally advanced esophageal cancer is being recommended as the new standard by the NCCN guidelines and the Chinese Society of Clinical Oncology guidelines (67). Neoadjuvant immunotherapy offers a novel option for neoadjuvant treatment of esophageal cancer, and preliminary clinical results indicate that it can lead to disease remission in some patients with esophageal cancer. Moreover, immunotherapy combined with chemotherapy effectively improves the PCR rate of esophageal cancer patients compared with chemotherapy alone without causing significant treatment-related adverse events. This combination is expected to prolong the survival of patients with locally advanced resectable esophageal cancer and become a crucial model in the neoadjuvant therapy of esophageal cancer in the future. Neoadjuvant immunotherapy for esophageal cancer is still in the exploratory stage, and several ongoing phase III studies in large-scale are expected to further investigate the safety, efficacy and optimal time-window for the administration of immunotherapy drugs.

Although immunotherapy is increasingly recognized as a crucial therapeutic strategy for the management of esophageal cancer treatment, clinical data reveal that only approximately 20%-40% of patients benefit from it. Therefore, understanding how the remaining patients can benefit from immunotherapy is an urgent clinical challenge. Many factors including PD-L1 expression, tumor neoantigen expression and delivery, related cellular signaling pathways, tumor microenvironment, epigenetic modifications have been confirmed to involved in the response to PD-1/PD-L1 blockade therapy (68). Regulatory T cells (Tregs) play a role in suppression of effector T cell response by secretion of IL-10, IL-35, and TGF-β. PD-1 expression upregulates conversion of naive CD4^+^ T cells to immunosuppressive Treg cells through inhibition of the mTOR-Akt signaling cascade. Aberrant cellular signal transduction including the PI3K/AKT pathway, WNT/β-catenin pathway, JAK/STAT/IFN-γ pathway, and MAPK pathway have also been proved to be major factors to immunotherapy resistance (69–71).

In addition, numerous findings have revealed in ESCC patients the instability of genome and alterations in epigenome (66, 67, 72–74), which led to significant heterogeneity among ESCC patients (75). A study from Cancer Hospital, Chinese Academy of Medical Sciences classified ESCC into four subtypes (cell cycle pathway activation, NRF2 oncogenic activation, immune suppression, and immune modulation) based on multi-omics data analysis. This is the first internationally to establish multi-omics-based classification for ESCC. In addition, they identified potential therapeutic targets/biomarkers that can be employed for diagnosis for each subtype, providing theoretical basis and novel strategies for achieving precision medicine for ESCC patients. Recently, our findings reveal a novel mechanism by which ESCC cells escape immune surveillance through TP63-suppressed interferon-STAT1 axis and cytotoxic CD8 T cells. And importantly, inhibition of TP63 enhance the efficacy of PD-1 mAb therapy in syngeneic mouse models.

Thus, to further improve clinical benefits, future efforts should be made to identify predictive biomarkers and target patients for immunotherapy, to explore factors that affect patient response to immune checkpoint-based therapy. In clinical managements, the cycles of neoadjuvant immunotherapy, the ways to surmount the primary and acquired resistance, as well as the maintenance of a durable efficacy of immunotherapy remain unavoidable challenges in the future.

## 6 Summary and outlook

Improvements have been made to increase R0 resection rates and PCR rate as well as survival outcomes with neoadjuvant chemotherapy combining ICB therapy for locally advanced ESCC. In addition, ICB therapy in an adjuvant setting has revolutionized the treatment landscape of ESCC. In the future, incorporation of immunotherapy in the preoperative neoadjuvant treatment, the optimal time-window for the administration of immunotherapy, multi-disciplinary treatment model and elucidation of pathogenic mechanism of ESCC will provide precise strategies for this type of cancer. In addition, further innovations in endoscopic, surgical resection and radiotherapy approaches as well as cell therapy such as CAR-T, TCR-T would potentially improve the outcomes of ESCC patients.

## Author contributions

DW: Conceptualization, Data curation, Funding acquisition, Investigation, Writing – original draft, Writing – review & editing. YJ: Funding acquisition, Writing – review & editing, Data curation. JF: Data curation, Writing – review & editing. HW: Data curation, Writing – review & editing. JC: Data curation, Investigation, Writing – review & editing. SK: Visualization, Writing – review & editing. Y-YJ: Funding acquisition, Investigation, Writing – review & editing. 
